# Comparative analysis between a low pathogenic and a high pathogenic influenza H5 hemagglutinin in cell entry

**DOI:** 10.1186/1743-422X-6-76

**Published:** 2009-06-10

**Authors:** Emily Rumschlag-Booms, Ying Guo, Jizhen Wang, Michael Caffrey, Lijun Rong

**Affiliations:** 1 Department of Microbiology and Immunology, College of Medicine, University of Illinois at Chicago, Chicago, Illinois 60612, USA; 2 Institute of Materia Medica, Peking Union Medical College and Chinese Academy of Medical Sciences, Beijing 100050, PR China; 3 Department of Biochemistry and Molecular Genetics, College of Medicine, University of Illinois at Chicago, Chicago, Illinois 60607, USA

## Abstract

Avian influenza viruses continue to threaten globally with pandemic potential. The first step in a potential pandemic is the ability of the virus to enter human cells which is mediated by the viral surface glycoprotein hemagglutinin (HA). Viral entry of influenza is dependent upon the processing of the HA_0 _polypeptide precursor protein into HA_1 _and HA_2 _which is mediated by host cellular proteases. The sequence of the cleavage site which is recognized by host proteases has been linked with pathogenesis of various influenza viruses. Here we examined the effects of cleavage site sequences between a highly pathogenic H5N1 strain and a low pathogenic H5N2 strain to determine their effects on viral entry. From this analysis we determined that at the level of viral entry, the only observed difference between the low and high pathogenic strains is their ability to be cleaved by host cellular proteases.

## Findings

Influenza A viruses have two glycoproteins on their surface, neuraminidase (NA) and hemagglutinin (HA). While NA is believed to be crucial in the budding process to release new viral particles from the host cell surface, HA is thought to be important in the entry of the virus, as this protein mediates binding to its receptor, sialic acid (SA) as well as fusion of the viral envelope with the endosomal membrane [1]. HA is synthesized as a single precursor polypeptide, HA_0_, which must be cleaved by host proteases into HA_1 _and HA_2 _in order to be biologically active. Cleavage is necessary for the virus to establish infection in the host as well as to spread within the host. The host enzymes responsible for this cleavage event are believed to correspond with the pathogenicity of the virus and are determined based on the cleavage site sequence [2-5]. The majority of HA subtypes posses a single arginine at their cleavage site which facilitates cleavage by trypsin, a protease mainly localized to the respiratory tract in humans and the gastrointestinal tract in birds. The restricted expression of these proteases correlates with the sites of localized infection for each host, linking them to limited spread through the host and therefore potentially lower virulence [4]. In contrast, highly pathogenic strains such as H5 and H7 influenza A viruses are believed to be more virulent than other HA subtypes as these viruses utilize substilin-like proteases to cleave HA_0 _[3,4,6-8]. This class of proteases is ubiquitously expressed throughout a variety of hosts including birds and humans. Due to its wide distribution, HA_0 _can be activated by a variety of cells and thus, can easily spread systemically. The consensus recognition site for this class of proteases, which includes furin, is R-X-K/R-R [4]. It is thought that the HAs from highly pathogenic strains have acquired these cleavage sequences through insertion mutations.

**Figure 1:**
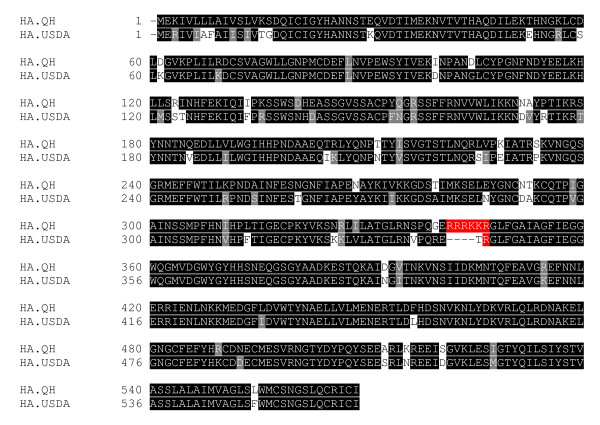
**Sequence alignment of uncleaved low pathogenic H5N2 HA USDA and high pathogenic H5N1 HA Qinghai (QH)**. Amnio acids implicated in cleavage of HA_0 _into HA_1 _and HA_2 _are highlighted in red.

In light of the current highly pathogenic H5N1 virus currently circulating, we sought to understand the differences of HA between a highly pathogenic H5N1 virus and a low pathogenic H5N2 virus in entry. Sequence alignment between these HAs reveals a homology of approximately 88% with the major difference at the HA_0 _cleavage site (Fig. 1). The H5N1 HA contains the sequence required by the substilin-like proteases (R-K-K-R), while the H5N2 HA carries a single arginine at this site [9]. We proposed that the major difference between the highly pathogenic HA and the low pathogenic HA at the entry level is their ability to be cleaved and activated by host cellular proteases.

Previously, we developed an HIV-based pseudotyping system and demonstrated that a highly pathogenic H5N1 recombinant virus can enter human-derived cell lines more efficiently than avian-derived cell lines [10]. Having determined the tropism of this highly pathogenic H5N1 virus [11], we wanted to compare the differences at the level of entry with a low pathogenic H5N2 virus [9] utilizing the aforementioned pseudotyping system. This pseudotyping system allows us to safely and specifically study the HA protein of influenza A viruses at the entry level by incorporating the HA gene into HIV virion particles and using them for transduction to the target cells. Briefly, human embryonic kidney 293 T cells were co-transfected using PEI (Invitrogen) with a pNL4.3.R-E- plasmid carrying a luciferase reporter gene [12,13] and a pcDNA3.1 plasmid carrying the appropriate HA gene. Producer cells were directly treated twenty-six and forty-six hours post-transfection with 100 U/mL of purified neuraminidase to facilitate release of viral particles produced. Forty-eight hours post-transfection viral particle containing supernatant was collected and 500 mL was used to transduce 293 T and A549 target cells. Forty-eight hours post-transduction, target cells were lysed and used to measure luciferase levels as an indication of viral entry.

**Figure 2:**
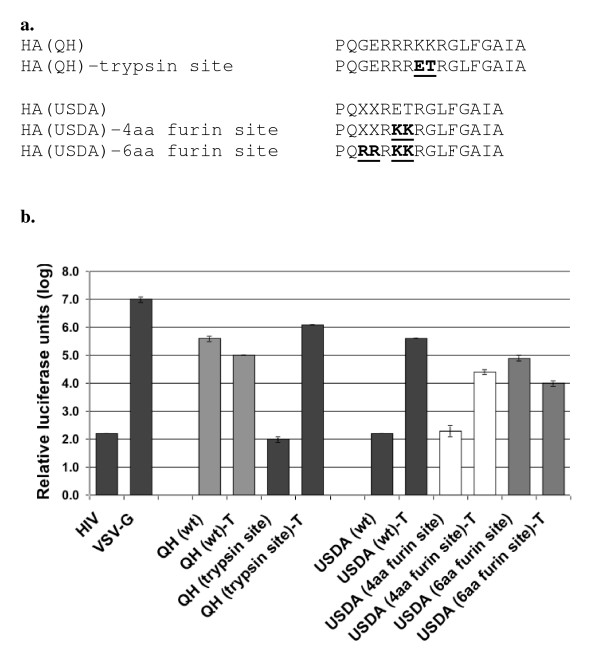
**Comparative analysis of HA_0 _cleavage site sequence in viral entry**. (A) Sequence alignment of site-direct mutagenesis in HA_0 _cleavage site sequences of HA USDA and HA QH. (B) Relative infectivity of pseudoviruses containing specified HA_0 _cleavage site sequences.

The HIV vector alone was used as a negative control as it does not carry a surface glycoprotein necessary to mediate entry. Luciferase levels for the HIV vector were comparable to the luciferase levels for cells alone (data not shown). VSV-G was used as a positive control as it is known that many cell types are susceptible to entry by this viral glycoprotein (Fig. 2b). Viral particles carrying the H5N2 USDA-HA (wt) gave luciferase levels comparable to the background levels, suggesting that the H5N2 HA was not able to mediate entry. Sequence analysis revealed that the H5N2 USDA-HA carries the trypsin cleavage site so we hypothesized that the lack of viral entry was due to the inability of the HA protein to be cleaved and activated by host cellular proteases. H5N2 viral particles requiring trypsin were collected and treated with 50 μg/mL of exogenous trypsin for thirty minutes at 37° to activate the HA protein. Both trypsin-treated H5N2 and H5N1 viruses were used to challenge several cell types from various species (Table 1). The H5N2 USDA-HA (wt), after trypsin treatment, was able to mediate entry at levels 1000-fold higher than the background. Trypsin treatment had little effect on the H5N1 QH-HA (wt). The cellular tropism for both the H5N2 trypsin-treated virus and the H5N1 virus were highly comparable in the nearly twenty cell types tested.

**Table 1:** Transduction of different cell lines

		RLUs	
Name of cell line	Cell type	H5N2 (USDA)-T^a, b^	H5N1 (QH)^c^
A549	Hu^d^, lung	1.6 × 10^5^	1.7 × 10^6^
NCI-H661	Hu, lung	1.1 × 10^5^	2.1 × 10^6^
HPAEC	Hu, lung	2.9 × 10^4^	7.6 × 10^4^
L2	Rat, lung	3.2 × 10^3^	5.1 × 10^3^
Lec 1	CH^e^, ovary	7.1 × 10^2^	2.5 × 10^3^
293T	Hu, kidney	5.0 × 10^6^	2.4 × 10^6^
A549	Hu, lung	2.2 × 10^5^	1.1 × 10^6^
HeLa	Hu, cervical carcinoma	1.8 × 10^4^	2.0 × 10^4^
QT6	Quail, fibrosarcoma	7.0 × 10^4^	3.9 × 10^4^
DF-1	Chicken, embryo	3.1 × 10^4^	4.4 × 10^4^
CHO	CH, ovary	7.0 × 10^3^	4.1 × 10^3^
Lec 1	CH, ovary	6.2 × 10^2^	1.6 × 10^3^
Vero E6	AGM^f^, kidney	2.8 × 10^3^	9.5 × 10^3^
MDBK	Cow, kidney	8.4 × 10^2^	2.3 × 10^3^
A549	Hu, lung	1.9 × 10^5^	4.7 × 10^5^
SAOS-2	Hu, bone	1.3 × 10^6^	6.0 × 10^4^
HepG2	Hu, liver	7.1 × 10^3^	3.1 × 10^4^
Huh 8	Hu, liver	1.3 × 10^7^	8.1 × 10^6^
Jurkat	Hu, T lymphocyte	1.4 × 10^3^	2.1 × 10^3^
A20	Hu, B lymphocyte	7.4 × 10^4^	2.6 × 10^4^
3T3	Mouse, kidney	8.9 × 10^2^	1.7 × 10^3^
RAW264.7	Mouse, macrophage	7.6 × 10^2^	1.6 × 10^3^
COS-7	AGM, kidney	8.6 × 10^3^	2.7 × 10^3^

a. HA (USDA)/HIV pseudovirions were treated with trypsin (50 μg/ml) for 30 min at 37°C prior to challenging the target cells.

b. T, Trypsin treatment.

c. HA (QH)/HIV pseudovirions were not treated with trypsin prior to challenging the target cells. These results are from reference [10] and are shown here for comparison.

d. Hu, Human.

e. CH, Chinese hamster.

f. AGM, African Green Monkey.

To further characterize the role of the cleavage site on HA function, the H5N1 QH-HA cleavage site of R-K-K-R was changed to that of the H5N2 USDA-HA, R-E-T-R, while the USDA-HA cleavage site was changed to that of H5N1 QH-HA (Fig. 2a). Pseudoviral particles carrying either of the two HA genes with their new cleavage sites were produced and used to challenge 293 T cells. Prior to challenge, aliquots of each virus were either treated with exogenous trypsin or left untreated. The QH-HA carrying the trypsin site gave luciferase levels at background without trypsin treatment, while treatment with trypsin restored infectivity to QH-HA (wt) levels (Fig. 2b). The USDA-HA carrying the four amino acid furin cleavage site was expected to be infectious without trypsin treatment, however, these viral particles were not infectious unless treated with exogenous trypsin.

Further sequence examination of the cleavage site of the QH-HA gene revealed that it carries the preferred six amino acid substilin-like cleavage site of R-R-R-K-K-R (Fig. 2a). This cleavage site was introduced into the USDA-HA gene using PCR site-directed mutagenesis. Viral particles produced carrying USDA-HA with the six amino acid cleavage site were infectious nearly 1000-fold over the background levels without trypsin treatment (Fig. 2b). Further treatment with trypsin had little to no effect on the infectivity of these viral particles.

## Conclusion

The data presented here takes aim at the differences between a highly pathogenic H5 HA and a low pathogenic H5 HA at the level of entry. It has been established that the cleavage site sequence of the HA_0 _protein is linked to pathogenicity, with highly pathogenic strains carrying the substilin-like cleavage sequence while low pathogenic strains carry the trypsin cleavage site sequence, however it is not known what other differences there are between these two types of HA proteins. Here we demonstrate that at the level of entry, the highly pathogenic H5N1 HA and the low pathogenic H5N2 HA have the same cellular tropism as long as the HA_0 _protein is activated by trypsin treatment if it does not carry the six amino acid substilin-like cleavage site. While highly pathogenic strains garner more attention based on their feared ability to spread from human to human, this study draws attention to low pathogenic strains which already have the capability for human-to-human transmission and need only alter their cleavage site sequence. Based on this data, low pathogenic influenza strains may threaten to become highly pathogenic strains if they acquire the necessary amino acids to be processed and activated by the substilin-like proteases.

## Competing interests

The authors declare that they have no competing interests.

## Authors' contributions

ERB, YG, JW, MC, and LR participated in the design of the study and drafted the manuscript. ERB, YG, and JW performed the experiments. All authors have read and approved the final manuscript.
